# Eye, Ear, Nose and Throat

**Published:** 1893-08

**Authors:** 


### SELECTIONS AND ABSTRACTS.

### EYE, EAR, NOSE AND THROAT.

EDITED BY DR. ARTHUR G. HOBBS, ASSISTED BY
DR. GEORGE BROWN.

### HOMATROPINE SUSCEPTIBILITY.

We have, in the *Medical News* of January, a case of homatropia susceptibility reported by Dr. George M. Gould. He used an eight-grain solution and did not exceed two drops. This resulted in great swelling of the lids. The cheeks for two inches below were puffed and discolored. In this regard I wish to report a case of poisoning from this drug. On February 10th Miss F. H. F. called at my office to consult me about her eyes. After asking her a few question I decided she had an error of refraction, and at once began to instill in her eyes a solution prepared the day previous, containing four grains of homatropinia hydrobromatis to two drachms of distilled water. This I dropped in her eyes every five minutes for half an hour. After wating one hour from the last drop instilled in her eyes, I called her in to test them. I noticed, as she came toward me, that her gait was unsteady; but being a child fourteen years old, and an exceedingly nervous one, I paid no attention to it; but when she failed to enunciate her letters with distinctness, I asked her if she felt badly; she replied that she felt slightly nauseated. Within ten minutes she lost complete control of her lower limbs, this being quickly followed by drowsiness. Pulse rapid and fluttering. I gave her a stimulant and one eighth of a grain of morphia. I then called in Dr. Young to assist me. He gave her the second dose of morphia one hour after the first. It was four hours before she was perfectly conscious, and at least six hours before she had good use of her limbs. On the following morning I found she had hypermetropia, $\frac{20}{120}$. The pupils remained dilated for three days. I was sure of my drug because I had used it the day previous, the same day, and also the following day without any other unfavorable symptoms. I can account for this unusual case only on the grounds of a peculiar idiosyncrasy.—*The American Practitioner and News, July*, 1893.

["Idiosyncrasy," although a meaningless word, "covers a multitude of sins" for us. Yet it does express that something in the thousandth individual which we know not of until we find it expressing itself, oftenest, too, when we are least expecting it. Perhaps these anomalous effects have occasionally followed the administration of all potent drugs, hence the rare but interesting cases reported above from homatropia.

Similar anomalous effects of cocaine occur not unfrequently, even as the result of very small doses. (See two cases in this number reported by Dr. Geo. Brown.) Yet we do not speak of one who evinces no effect whatever from enormously large doses of cocaine as idiosyncratic. A number of these latter cases has come under my observation.—ED.]

---

### TWO CASES OF COCAINE SUSCEPTIBILITY.

No. 1.—Young man, florid, stoutly built, healthy and robust, farmer. Operation for stricture of lachrymal duct. Had used about one-half of the contents of small dropper, which held about thirty-five drops of 4 per cent. cocaine solution. While waiting a moment, attention was called to the patient by his drawing a deep, sighing respiration, then falling to the floor in a dead faint. Gave half glass of whisky, and in a few moments he recovered, but was in a dazed condition for half an hour.

No. 2.—Young lady, actress, delicate, anæmic looking girl, suffering from an attack of acute tonsillitis. Sprayed about fifteen drops of a 2 per cent. solution on tonsils and pharynx, preparatory to the application of a thirty grain solution of nitrate of silver to the tonsils. Patient immediately suffered great distress, evinced by difficulty of breathing, and said she felt that she was "smothering to death." Gave her glass of sherry, and made application to tonsils.

She appeared to get all right, but upon walking to waiting room fell in a faint. Gave another glass of sherry, bathed face with ice water, and in a few moments she recovered sufficiently to go home, but was quite ill and nervous for eight or ten hours afterwards.

GEORGE BROWN.

---

### THE FUNCTIONS OF THE TONSILS.

Gulland (*Edinburgh Medical Journal,* November, 1891). The faucial, lingual and pharyngeal tonsils are important factors in the reproduction of leucocytes. The leucocytes so formed may be carried into the general circulation by lymphatics, may become fixed as stationary cells in the tonsils or may pass through the epithelial cells lining the crypts.

On the surface of the tonsil they act as ready absorbents for micro-organisms, arrest their progress into the general system and thus protect the body from them. The faucial and lingual tonsils and the infiltration of leucocytes under the velum act as guarding zones between the mouth and alimentary canal, and the pharyngeal and tubal tonsils and leucocyte infiltration of upper palatal surface form a ring at upper extremity of respiratory tract. During health the tonsils do not act as absorbents, the entrance of any foreign body into the tonsil being prevented by the constant outward stream of leucocytes. During disease the regular productiun of leucocytes is interfered with, their outward flow is interrupted and septic micro-organisms may enter tonsilar tissue, causing local or general infection.—*Brooklyn Medical Journal.*

[The danger of infection from a pathological condition of either or all of the three tonsils is probably confined to the follicular form of inflammation where the products of the inflammatory process are confined in the crypts and degenerate, consequently either local or general infection may result through lymphatic absorption.—ED.]

